# The first non‐radiographic axial spondyloarthrits with COVID‐19

**DOI:** 10.1002/iid3.448

**Published:** 2021-05-12

**Authors:** Wassim Saikali, Suzanne Gharib

**Affiliations:** ^1^ Rheumatology and Pulmonary Clinic Beckley West Virginia USA; ^2^ The Rheumatology Group South Charleston West Virginia USA

**Keywords:** CoV‐SARS‐2, non‐radiographic axial spondyloarthritis

## Abstract

**Background:**

Case report of a 21‐year‐old female developing non‐radiographic axialspondyloarthritis in the setting of a preceding severe acute respiratory syndrome coronavirus 2 (SARS‐CoV‐2) infection. We felt this was a unique case as some cases of psoriatic arthropathy and reactive arthropathy but none, to our knowledge of non‐radiographic axial spondyloarthritis.

**Case Presentation:**

Twenty one‐year‐old female presented in June 2020 with inflammatory symptoms with general work‐up per primary care provider being negative. Upon further work‐up per Rheumatology, felt to have inflammatory back pain with X‐rays of SI joints showing grade II sclerosis. Further work‐up including magnetic resonance imaging was positive demonstrating bone edema and erosions of SI joint. This prompted further investigation and she had had some vague symptoms of SARS‐CoV‐2 several months prior including loss of smell, fatigue, and malaise. SARS‐CoV‐2 semiquanitative antibodies where done that where high positive. She was treated with cetrolizumab and had prompt improvement over the course of the next week.

**Discussion and Conclusion:**

Mechanism of SARS‐CoV‐2 triggering autoimmunity is still unknown at this time. However, it is important that insights be gained into the type of disease that can be triggered by this infection. As the pandemic continues to rage on, Rheumatologist need to be increasing aware that patients presenting with various forms of inflammatory arthritis may be triggered by an antecedent SARS‐CoV‐2 infection. Following these cases may help to determine how they differ from other forms of inflammatory arthropathy.

## Graphical abstract

A case of non radioghraphic ankylosing spondyloarthritis after coronavirus disease 2019 exposure.

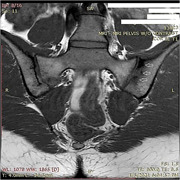

## 1 BACKGROUND

In late December 2019, several health facilities reported clusters of patients with pneumonia of unknown cause that where linked epidemiologically to a seafood and wet animal wholesale market in Wuhan, China. By January 7, 2020, Chinese CDS confirmed isolation of novel coronavirus. By March 11, 2020, coronavirus disease 2019 (COVID‐19) was announced to be a global pandemic by the WHO. The rest, after that, is history. The pandemic, of a virus whose name is now officially severe acute respiratory syndrome coronavirus 2 (SARS‐CoV‐2), has raged on since and there continues to be a wide array of findings that are being seen daily in clinics across the country. Given the links to autoimmune and autoinflammatory disease that have been emerging, the field of Rheumatology is being brought into the front and center of this pandemic and as survivors emerge, so do new inflammatory diseases that appear to be linked to the SARS‐CoV‐2 infection.

Review of the literature that currently exists has found several casReview of the literature that currently exists has found several case reports of reactive arthritis, rheumatoid arthritis and psoriatic arthritis. However, to our knowledge, at the time of writing this article, there were no cases of ankylosing spondylitis or non‐radiographic axial spondyloarthritis. This case report is of a patient presenting with just that in September 2020. As these cases emerge in Rheumatology clinics nationwide, it is going to be increasingly important that the information is shared as this is likely to be a significant issue that arises in the post‐COVID population. Aside from the toll of acute injury, we are beginning to discover the long term implications of SARS‐CoV2 infection.

## 2 CASE PRESENTATION

Twenty one‐year‐old female presented to her primary care physician in June 2020 with complaints of diffuse joint pain with particular focus of hip and knee pain. She also reported low grade fever and mild diarrhea. Work up was done at that time including ANA, RF, ASO titer, Ferritin, all of which were normal. There were mild elevations of acute phase reactants, specifically ESR 31 mm/h (0–20 mm/h) and CRP 13 mg/L (0–10 mg/L).

Due to nonconclusive work‐up and persistent symptoms, patient was referred to Rheumatology in September 2020. Her primary complaints at first visit were back pain that was worse in the morning and stiffness in the back and knees lasting several hours. Upon further questioning, she reported symptoms where worse at rest and improved with activity. She did not have fever, upper respiratory symptoms or diarrhea at that time. On exam, she had sacroiliac joint tenderness on the left side and left knee had mild effusion and joint tenderness. Based on presentation, she was felt to have early inflammatory arthropathy, likely reactive as she had diarrheal illness several weeks before presentation. A course of NSAID was given with minimal improvement.

Over the next few weeks, her symptoms worsened and in December 2020, returned to clinic with severe back pain limiting mobility and function. Stiffness in the morning reported to last up to 4 h. Pertinent positives on exam was consistent with limited mobility in lumbar spine as well as tenderness in the bilateral SI joints.

**Figure 1:**
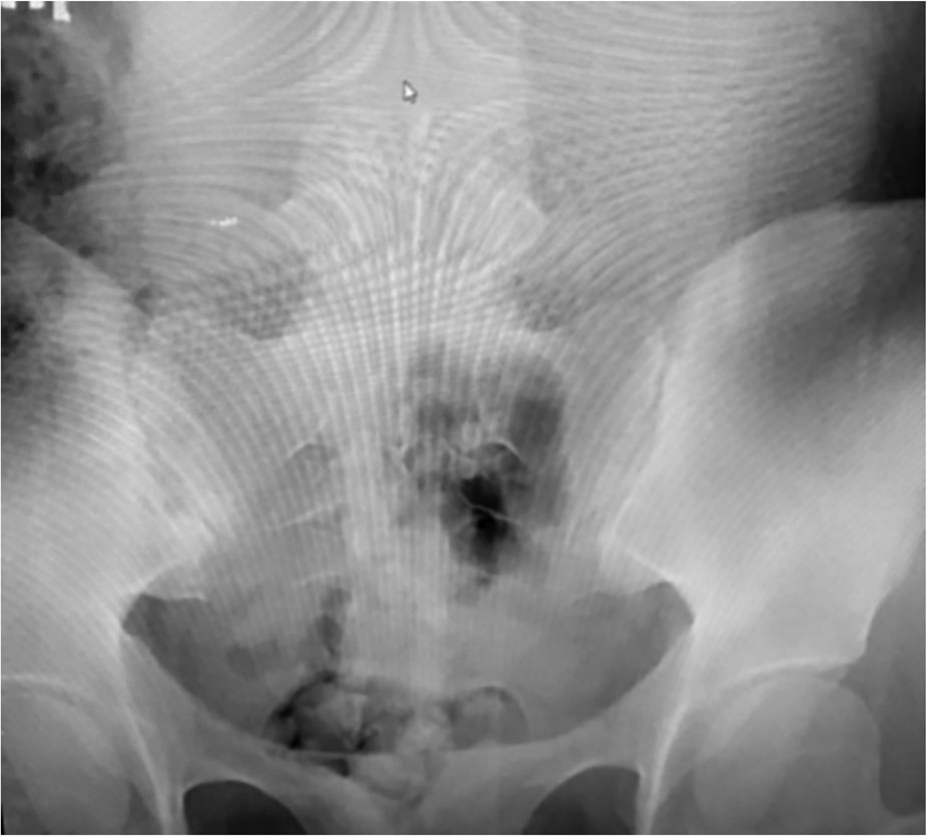
Early sclerosis bilateral SI joints

**Figure 2:**
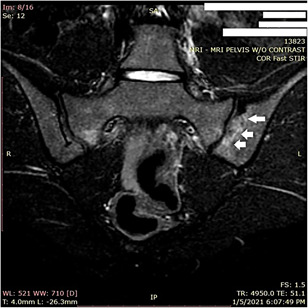
Bone edema on the left SI joint (arrows)

**Figure 3:**
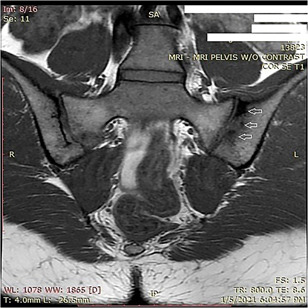
Bone marrow erosions on the left SI joint (arrows)

Based on new findings, HLA B27 was checked and was negative. Imaging was obtained of SI joints that demonstrated Grade II sclerosis, which was felt to be abnormal for her age. Based on abnormality on X‐ray (Figure 1), magnetic resonance images were obtained at that time that demonstrated bone marrow edema bilaterally on T2 STIR imaging, left greater than right (Figure 2) and erosions of the SI joint on left on T1 weighted imaging (Figures 3 and 1).

Accordingly, she was diagnosed with non‐radiographic axial spondyloarthritis based on clinical presentation and imaging criteria and absence of clear findings on plain film to meet NY criteria for Ankylosing Spondylitis. She was subsequently started on a TNF‐alpha inhibitor, certolizumab and had impressive improvement in both function and pain within 2 weeks.

Due to concern about the rapidity of symptom progress, further history was elicited regarding her preceding infection after diagnosis was established. Initially, she had reported diarrhea and low grade fever but upon further questioning, she reported loss of taste at that time as well as fatigue and malaise. She had not been tested for SARS‐CoV‐2 as part of her initial work up. However, given the history of those symptoms, SARS‐CoV‐2 antibody semi quantitative testing was done on January 8, 2021 and found to be high positive, 175 units/ml (normal range <0.8 unit/ml), which was an indication of old infection and natural immunity as patient had not received SARS‐CoV‐2 vaccine.

## 3 DISCUSSION

In the medical literature, it is a well known fact that AS and related conditions, such as reactive arthritis, can be triggered by infections in a susceptible host.^1^ The course that disease takes is highly variable and can range from self limited localized arthritis to chronic progressive disease.

Notably, the increase in inflammatory cytokines during SARS‐CoV‐2 has a major role in inciting and promoting the immunologic response that has been observed as a serious complication of acute SARS‐CoV‐2 infection. Clinically, this has been observed to lead to a variety of outcomes in predisposed patients. However, the exact process of how that happens remains poorly defined. In particular, the severity of the antecedent infection seems to have little, it any impact, on the severity of the downstream immunologic responses observed in patients. The patient presented in this case had a GI presentation associated with SARS‐CoV‐2. To date, GI involvement with SARS‐CoV‐2 has been perceived as primarily a secondary site for infection, related to the expression of angiotensin‐converting enzyme‐2 in the gastrointestinal tract.^2^ That being said, it has not been particularly associated with severe symptomology of acute infection.

The observed percent of patients with acute SARS‐CoV‐2 who have GI symptoms, appears to be about 12% as of the time of this publication.^3^ It raises several questions as to how that triggers immune response. Could it be that infection triggers a change in the microbiome leading to an immunologic response? Is this a case of reactive arthritis that rapidly progressed to nonradiographic axial spondyloarthritis or is this a case of early AS that was accelerated by acute SARS‐CoV‐2 infection? Clearly, there is an increasing recognition among Rheumatologists that the spondlyoarthritides are more of a spectrum then truly individual diseases and this case certainly highlights the pitfalls of naming these as finite entities with no observable overlap.

Clearly, this case also illustrates the importance of autoimmune and autoinflammatory diseases being triggered by SARS‐CoV‐2 infections. It will certainly help to better understand the pathophysiology of SARS‐CoV‐2 infections. Conversely, a better understanding of how these diseases emerge more acutely may also lend insight as to identifying risk factors for rapidly worsening axial spondylarthritides which remain difficult to identify in early phases.

In the medical literature, it is a well known fact that AS and related conditions, such as reactive arthritis, can be triggered by infections in a susceptible host.^1^ The course that disease takes is highly variable and can range from self limited localized arthritis to chronic progressive disease.

Notably, the increase in inflammatory cytokines during SARS‐CoV‐2 has a major role in inciting and promoting the immunologic response that has been observed as a serious complication of acute SARS‐CoV‐2 infection. Clinically, this has been observed to lead to a variety of outcomes in predisposed patients. However, the exact process of how that happens remains poorly defined. In particular, the severity of the antecedent infection seems to have little, it any impact, on the severity of the downstream immunologic responses observed in patients. The patient presented in this case had a GI presentation associated with SARS‐CoV‐2. To date, GI involvement with SARS‐CoV‐2 has been perceived as primarily a secondary site for infection, related to the expression of angiotensin‐converting enzyme‐2 in the gastrointestinal tract.^2^ That being said, it has not been particularly associated with severe symptomology of acute infection.

The observed percent of patients with acute SARS‐CoV‐2 who have GI symptoms, appears to be about 12% as of the time of this publication.^3^ It raises several questions as to how that triggers immune response. Could it be that infection triggers a change in the microbiome leading to an immunologic response? Is this a case of reactive arthritis that rapidly progressed to nonradiographic axial spondyloarthritis or is this a case of early AS that was accelerated by acute SARS‐CoV‐2 infection? Clearly, there is an increasing recognition among Rheumatologists that the spondlyoarthritides are more of a spectrum then truly individual diseases and this case certainly highlights the pitfalls of naming these as finite entities with no observable overlap.

Clearly, this case also illustrates the importance of autoimmune and autoinflammatory diseases being triggered by SARS‐CoV‐2 infections. It will certainly help to better understand the pathophysiology of SARS‐CoV‐2 infections. Conversely, a better understanding of how these diseases emerge more acutely may also lend insight as to identifying risk factors for rapidly worsening axial spondylarthritides which remain difficult to identify in early phases.

An acknowledged limitation of our case is the absence of testing for acute SARS‐CoV‐2 at initial presentation. Obviously, other infections could have triggered similar findings although that seems unlikley as evidence of anticedent SARS‐CoV‐2 was seen based on antibody testing and no evidence of other infections was detected.

In conclusion, this case adds to the medical literature in terms of diseases triggered by SARS‐CoV‐2. Despite the devastation that this pandemic has wrecked on the medical community at large, the insights that can be gained by learning how it affects the immune system may give real depth going forward to the links between infectious disease and autoimmune/autoinflammatory conditions. This may in turn open pathways to earlier diagnosis and intervention.

## Data Availability

The data that support the findings of this study are available from the corresponding author upon reasonable request.
